# Construction of a restriction-less, marker-less mutant useful for functional genomic and metabolic engineering of the biofuel producer *Clostridium acetobutylicum*

**DOI:** 10.1186/s13068-016-0432-2

**Published:** 2016-02-02

**Authors:** Christian Croux, Ngoc-Phuong-Thao Nguyen, Jieun Lee, Céline Raynaud, Florence Saint-Prix, Maria Gonzalez-Pajuelo, Isabelle Meynial-Salles, Philippe Soucaille

**Affiliations:** LISBP, INSA, University of Toulouse, 135 Avenue de Rangueil, 31077 Toulouse Cedex, France; College of Life Sciences and Biotechnology, Korea University, Seoul, South Korea; Metabolic Explorer, Saint-Beauzire, France

**Keywords:** *Clostridium acetobutylicum*, *upp*, *Cac*824I, 5-FU, Gene deletion, Gene replacement, FRT, FLP, *upp* gene

## Abstract

**Background:**

*Clostridium acetobutylicum* is a gram-positive, spore-forming, anaerobic bacterium capable of converting various sugars and polysaccharides into solvents (acetone, butanol, and ethanol). The sequencing of its genome has prompted new approaches to genetic analysis, functional genomics, and metabolic engineering to develop industrial strains for the production of biofuels and bulk chemicals.

**Results:**

The method used in this paper to knock-out or knock-in genes in *C. acetobutylicum* combines the use of an antibiotic-resistance gene for the deletion or replacement of the target gene, the subsequent elimination of the antibiotic-resistance gene with the flippase recombinase system from *Saccharomyces cerevisiae*, and a *C. acetobutylicum* strain that lacks *upp*, which encodes uracil phosphoribosyl-transferase, for subsequent use as a counter-selectable marker. A replicative vector containing (1) a pIMP13 origin of replication from *Bacillus subtilis* that is functional in *Clostridia,* (2) a replacement cassette consisting of an antibiotic resistance gene (*MLS*^*R*^) flanked by two FRT sequences, and (3) two sequences homologous to selected regions around target DNA sequence was first constructed. This vector was successfully used to consecutively delete the *Cac*824I restriction endonuclease encoding gene (*CA_C1502*) and the *upp* gene (*CA_C2879*) in the *C. acetobutylicum* ATCC824 chromosome. The resulting *C. acetobutylicum* Δ*cac1502*Δ*upp* strain is marker-less, readily transformable without any previous plasmid methylation and can serve as the host for the “marker-less” genetic exchange system. The third gene, *CA_C3535*, shown in this study to encode for a type II restriction enzyme (*Cac*824II) that recognizes the CTGAAG sequence, was deleted using an *upp*/5-FU counter-selection strategy to improve the efficiency of the method. The restriction-less marker-less strain and the method was successfully used to delete two genes (*ctfAB*) on the pSOL1 megaplasmid and one gene (*ldhA*) on the chromosome to get strains no longer producing acetone or l-lactate.

**Conclusions:**

The restriction-less, marker-less strain described in this study, as well as the maker-less genetic exchange coupled with positive selection, will be useful for functional genomic studies and for the development of industrial strains for the production of biofuels and bulk chemicals.

## Background

In recent years, *Clostridium acetobutylicum* ATCC824 has been of interest in the postgenomic era due to the complete sequencing and annotation of its genome [1], supplying a wealth of information regarding its protein machinery. This global knowledge has prompted new approaches to genetic analysis, functional genomics, and metabolic engineering in order to develop industrial strains for the production of biofuels and bulk chemicals.

To this end, several reverse genetic tools have been developed for *C. acetobutylicum* ATCC 824, including a gene inactivation system based on non-replicative [2, 3] and replicative plasmids [4–7] and the group II intron gene inactivation system [8, 9]. Among these methods, only the method developed by Al-Hinai et al. [5] allows for *in frame* deletions and/or the introduction of genes at their normal chromosomal context without an antibiotic marker remaining. This system is made of two parts. The first part is a replicative vector containing (1) a pIMP13 origin of replication from *Bacillus subtilis* functional in *Clostridia,* (2) a replacement cassette consisting of an antibiotic resistance gene (*Th*^*R*^) flanked by two FRT sequences, (3) two sequences homologous to the selected regions around the target DNA sequence, and (4) a codon-optimized *mazF* toxin gene from *Escherichia coli* under the control of a lactose-inducible promoter from *Clostridium perfringens* to allow for the positive selection of double-crossover allelic exchange mutants. The second part is a plasmid system with inducible segregational instability, enabling efficient deployment of the FLP-FRT system to generate marker-less deletion or integration mutants.

In 2006, our group patented a marker-less, in-frame deletion method [10] similar to the two-part method published by Al-Hinai et al. [5] in 2012. The first part of our method is based on the same replicative plasmid and the same replacement cassette, but it uses the uracil PRTase *upp*/5-fluorouracil (5-FU) system as a counter-selection strategy. The second part is based on a plasmid carrying (1) the FLP-FRT system to generate marker-less deletion and (2) the uracil PRTase *upp*/5-FU system to select for the plasmid loss after marker excision. This method was successfully used by the Metabolic Explorer Company to develop and patent an industrial recombinant strain of *C. acetobutylicum* for *n*-butanol production. As this method has not been described in detail and to make it available to and usable by the scientific community, we report how this method was developed and its use to create a restriction-less, marker-less strain of *C. acetobutylicum*. We show that this strain lacking *upp* (*CA_C2879*, encoding the uracil–phosphoribosyl-transferase), *CA_C1502* encoding *Cac*824I and *CA_C3535* encoding *Cac*824II (the second type II restriction enzyme) can be transformed by non-methylated DNA at very high efficiency and can be used for rapid gene knock-in and knock-out using the *upp*/5-FU counter-selectable system for both functional genomic and metabolic engineering of *C. acetobutylicum*. This strain and the method were further used to delete three genes *ctfAB* and *ldhA* to create strains no longer producing acetone and lactate, respectively.

## Results

### MGCΔ*cac1502* strain, a *C. acetobutylicum* strain that is transformable without previous in vivo plasmid methylation

*Cac*824I, the type II restriction endonuclease encoded by *CA_C1502*, is a major barrier to the electrotransformation of *C. acetobutylicum* with *E. coli*–*C. acetobutylicum* shuttle vectors [11]. The *Cac*824I restriction endonuclease recognition sequence 5′-GCNGC3′, where N can be any nucleotide, occurs infrequently in *C. acetobutylicum* DNA because of the high A + T DNA content (72 % A + T), but the sequence occurs frequently in *E. coli* plasmids. No methyltransferase that can be used in vitro to protect DNA from restriction by *Cac*824I is commercially available. Prior to the transformation of *C. acetobutylicum*, shuttle plasmids have to be methylated in vivo by transformation into *E.coli* ER2275 (pAN1) expressing the *Bacillus subtilis* phage φ3TI methyltransferase, which protects the shuttle plasmids from digestion by the clostridial endonuclease *Cac*824I [11]. This step is time consuming and may be a drawback if the genes to be transferred to *C. acetobutylicum* are toxic when expressed in *E. coli*. Therefore, a *C. acetobutylicum* strain deficient for this particular restriction system would be valuable for efficient electrotransformation without previous treatment of the plasmid to be transformed.

**Fig. 1:**
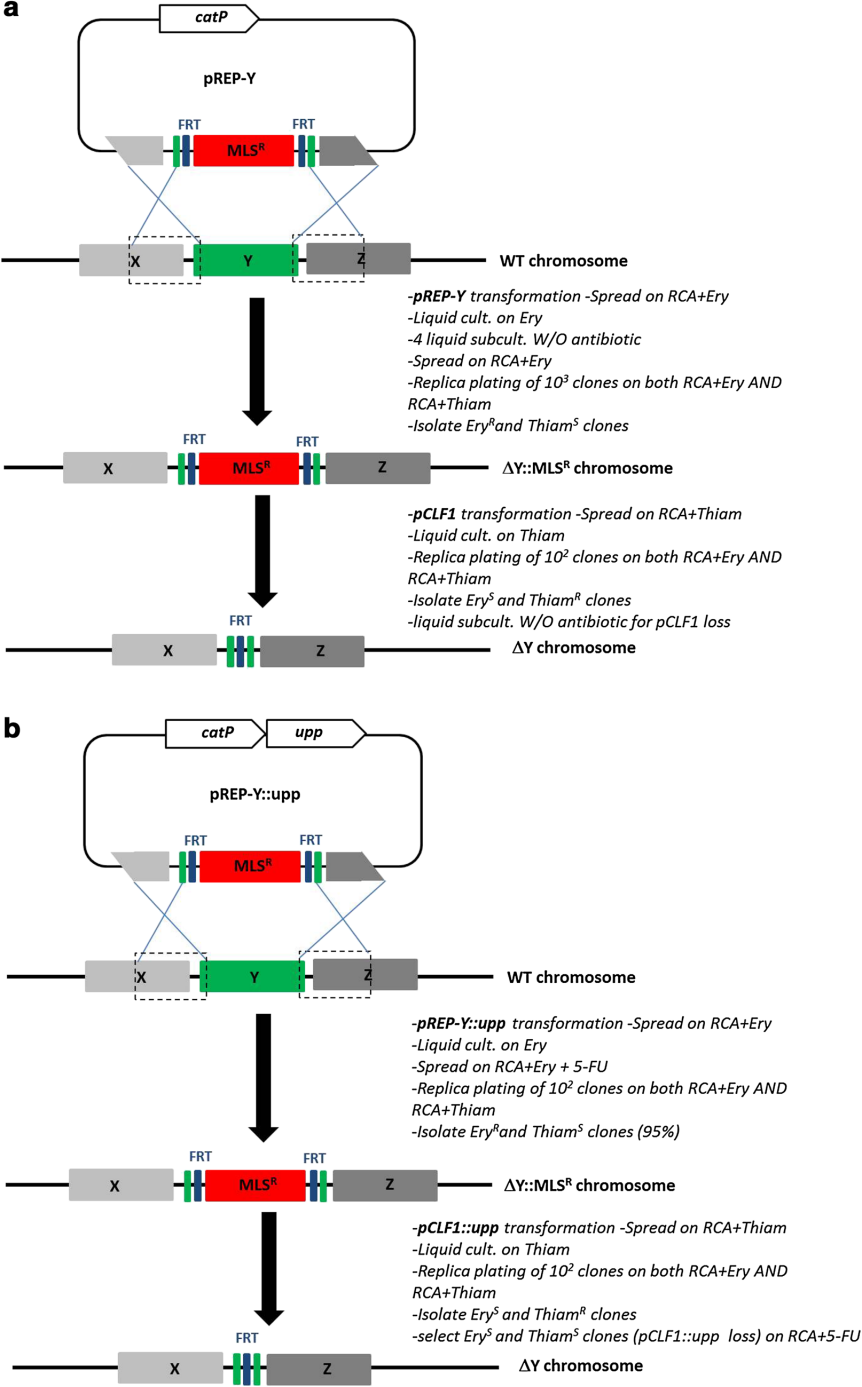
General diagram representing gene replacement via allelic exchange at the *Y* locus, and excision of the MLS^R^ marker by the FLP recombinase to create an unmarked *Y* deletion mutant. The *boxed regions of X* and *Z genes* represent approximatively the regions of homology incorporated into the replicative plasmid used for the double-crossover event (~ 1 kbp each). **a** Initial strategy used for the construction of the MGC*Δcac1502* and MGC*Δcac1502Δupp* strains, **b** counter-selection strategy with the 5-FU/*upp* system used for the construction of the MGC*Δcac1502ΔuppΔcac3535* strain

**Fig. 2:**
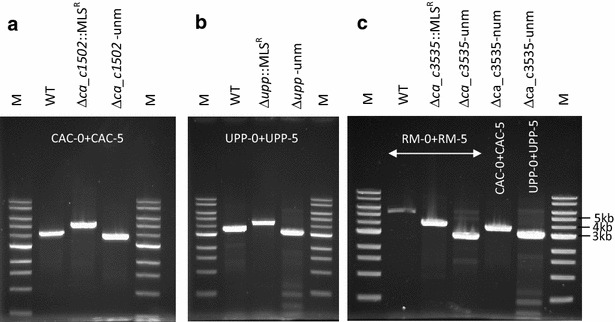
Gene replacement via allelic exchange at the *ca_c1502, upp* and *ca_c3535* loci. PCR confirmation of the different double-crossover deletion mutants using external primers annealing to the chromosome upstream and downstream of each deletion cassette used in the different pREP plasmids: **a** Δ*ca_c1502* deletion mutants with CAC-0 + CAC-5 primers, **b** Δ*upp* deletion mutants with UPP-0 + UPP-5 primers, **c** Δ*ca_c3535* deletion mutants with RM-0 + RM-5 primers (*lanes 1*, *2* and *3*). For each experiment, *lanes 2* and *3* refer to before and after excision of the MLS^R^ marker by the FLP recombinase, respectively, giving finally an unmarked deletion mutant (Δ-unm). The previous unmarked deletions in the final Δ*ca_c1502* Δ*upp* Δ*ca_c3535* were confirmed with CAC-0 + CAC-5 (**c**, *lane* *4*) and UPP-0 + UPP-5 (**c**, *lane* *5*) primers. *Lane M*, 1 kb DNA ladder (0.5–10 kb) (NEB)

To delete the *Cac*824I encoding gene, the first step is the construction of a shuttle vector carrying the replacement cassette. The *CA_C1502* replacement cassette was cloned into the *Bam*HI site of the pCons2-1 and pCIP2-1 to generate the pREPcac15 and pCIPcac15 plasmids, respectively. The difference between these two plasmids is the origin of replication. The pREPcac15 contains a pIMP13 origin of replication from *B. subtilis* (rolling circle mechanism) functional in *Clostridia*, whereas pCIPcac15 contains the origin of replication of the pSOL1 megaplasmid (θ replication mechanism). The pREPcac15 and pCIPcac15 plasmids were methylated in vivo in *E.coli* ER2275 (pAN1) and were used to transform *C. acetobutylicum* ATCC824 by electroporation. After selection on plates for clones resistant to erythromycin at 40 µg/ml, one colony of each transformant was cultured for 24 h in liquid SM with erythromycin and was then subcultured four times in liquid 2YTG medium without antibiotic (Fig. 1a). To select integrants that lost the pREPcac15 or pCIPcac15 plasmids, 10^3^ erythromycin resistant clones were replica plated on both RCA with erythromycin and RCA with thiamphenicol at 50 µg/ml. Whereas several colonies resistant to erythromycin and sensitive to thiamphenicol were obtained with pREPcac15 transformants, no such colonies were obtained with the pCIPcac15 transformants, which indicates that the θ replication mechanism of pCIPcac15 is less favorable for promoting double-crossover in *C. acetobutylicum* than a rolling circle mechanism. The genotype of clones with the desired phenotype was checked by PCR (polymerase chain reaction) analysis (Fig. 2a). The Δ*cac1502::mls*^*R*^ strain, which had lost the pREPcac15, was isolated. This strain was transformed with the pCLF1 plasmid expressing the *FLP1* gene of *S. cerevisiae* encoding for the FLP recombinase. The expression of *FLP1* was under the control of the promoter and RBS (ribosome binding site) from the thiolase gene from *C. acetobutylicum*. After transformation and selection for resistance to thiamphenicol at 50 µg/ml, one colony was cultured in liquid SM with thiamphenicol. One hundred thiamphenicol resistant clones were replica plated on both RCA with erythromycin and RCA with thiamphenicol. The genotype of the clones with erythromycin sensitivity and thiamphenicol resistance was checked by PCR analysis with primers CAC 0 and CAC 5 (Fig. 2a). Two successive 24-h liquid cultures of the Δ*cac1502* strain were conducted in the absence of antibiotics to remove pCLF1. The Δ*cac1502* strain that lost pCLF1 was isolated according to its sensitivity to both erythromycin and thiamphenicol. This strain was called MGCΔ*cac1502*.

The efficiency of transformation of this strain with methylated and unmethylated pCons2.1 plasmid was evaluated and compared to the wild type strain. Both strains can be transformed with methylated pCons2.1 with similar efficiency, but only MGCΔ*cac1502* can be transformed efficiently with unmethylated DNA (Table 1).

**Table 1:** Transformation efficiencies of *C. acetobutylicum* ATCC824 and MGC*Δcac1502* for unmethylated and methylated pCons2.1

	*C. acetobutylicum* ATCC824	MGC*Δcac1502*
Unmethylated pCons2.1	0	0.79 (±0.24) × 10^4^
Methylated pCons2.1	0.46 (±0.11) × 10^4^	0.58 (±0.18) × 10^4^

Values are expressed in number of transformants per μg DNA

Mean values and standard deviations from three independent experiments are given

25 ng pCons2.1 was used in each experiment

The following deletions described in this manuscript were conducted in this strain without previous in vivo plasmid methylation.

### Construction of the MGCΔ*cac1502*Δ*upp* strain: the first marker-free *C. acetobutylicum* strain with two deleted genes

To develop a positive screening of integrants, we used the “*upp*/5-FU as counter selection marker” system. The *C. acetobutylicumupp* gene (*CA_C2879*) encodes uracil phosphoribosyl-transferase (UPRTase), which catalyzes the conversion of uracil into UMP, thus allowing the cell to use exogenous uracil [12]. The pyrimidine analog 5-fluoro uracil (5-FU) can be converted by UPRTase into 5-fluoro-UMP, which is metabolized into 5-fluoro-dUMP, an inhibitor of thymidylate synthetase, toxic for the cell. The use of the *upp* expression cassette as a counter-selection marker is linked to the construction of a *C. acetobutylicum* strain deleted for the *upp* gene, thus resistant to 5-FU.

To delete *upp*, the *upp* replacement cassette was cloned into the *Bam*HI site of pCons2-1 to generate the plasmid pREPupp. The plasmid pREPupp was used to transform the MGCΔ*cac1502* strain by electroporation without previous in vivo methylation. After selection on plates for clones resistant to erythromycin at 40 µg/ml, one colony was cultured for 24 h in liquid SM with erythromycin and was then subcultured in liquid 2YTG medium without antibiotic (Fig. 1a). To select integrants having lost the pREPupp plasmid, 10^3^ erythromycin resistant clones were replica plated on both RCA with erythromycin and RCA with thiamphenicol. The genotype of the clones resistant to erythromycin and sensitive to thiamphenicol was determined by PCR analysis (Fig. 2b). The MGCΔ*cac1502*Δ*upp::mls*^*R*^ strain that lost pREPupp was isolated. When the resistance to 5-FU was analyzed, we showed that this strain was resistant to up to 1 mM 5-FU compared to 50 µM for the MGCΔ*cac1502* strain. This strain was then transformed with the pCLF1 plasmid, and selection of MGC Δ*cac1502*Δ*upp* strain with sensitivity to both erythromycin and thiamphenicol was performed, as previously described for the MGCΔ*cac1502* strain (Fig. 2b).

### Deletion of the *CA_C3535* gene in the MGCΔ*cac1502*Δ*upp* strain using the *upp*/5-FU system as a counter-selectable marker for the loss of plasmid

The *CA_C3535* gene encodes *Cac*824II, a potentially bi-functional enzyme carrying both a type II restriction endonuclease and methylase activities. To delete *CA_C3535*, the *CA_C3535* replacement cassette was cloned into the *Bam*HI site of the pCons::upp to generate the plasmid pREPcac3535::upp. The plasmid pREPcac3535::upp was used to transform the *C. acetobutylicum* MGCΔ*cac1502*Δ*upp* strain by electroporation without previous in vivo methylation.

After plate selection for clones resistant to erythromycin at 40 µg/ml, 100 transformants were replica plated on RCA with erythromycin, RCA with thiamphenicol and RCA with 5-FU at 400 µM (Fig. 1b). All transformants were resistant to erythromycin and thiamphenicol and were sensitive to 5-FU compared to the parental strain, which was resistant to 5-FU. This result demonstrates that the expression of the *upp* gene carried by pREPcac::upp confers sensitivity to 5-FU.

**Fig. 3:**
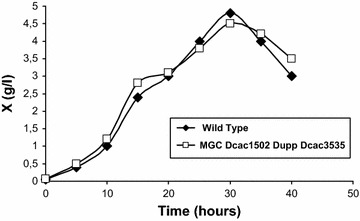
Growth curves of *C. acetobutylicum* ATCC824 and MGCΔ*cac1502* Δ*upp* Δ*cac3535* at pH 4.5 in SM medium

To select for Δ*cac3535::Em*^*R*^ integrants that lost the pREPcac3535::upp plasmid, erythromycin- and 5-FU-resistant clones were selected on RCA plates containing erythromycin and 5-FU from 100 µl of a liquid culture of the MGCΔ*cac1502*Δ*upp* (pREPcac3535::upp) strain. Approximately 500 colonies were obtained, and 100 of them were replica plated on both RCA with erythromycin and RCA with thiamphenicol. Most of the clones (95 %) were resistant to erythromycin and sensitive to thiamphenicol. Four clones were checked by PCR analysis (Fig. 2c) All four clones had the correct phenotype, and one of the clones was selected as the MGC Δ*cac1502*Δ*upp*Δ*cac3535::mls*^*R*^ strain. This strain was then transformed with pCLF::upp, a derivative of the pCLF1 plasmid that also carries the *upp* gene, in order for the positive selection of plasmid loss after the excision of the *mls*^*R*^ marker. After the first selection of clones resistant to thiamphenicol and sensitive to erythromycin, a second selection of clones resistant to 5-FU and sensitive to thiamphenicol was performed to obtain the MGC Δ*cac1502*Δ*upp*Δ*cac3535* strain that was control by PCR (Fig. 2c) for the presence of all the marker-less deletions. Finally, when compared to *C. acetobutylicum* ATCC824 wild type, the growth of the restriction-less marker-less strain in MS medium at pH 4.5 (Fig. 3) was shown to be unaffected by the different deletions.

The unmethylated pCons2.1 plasmid was used to evaluate the transformation efficiency of the MGCΔ*cac1502* and the MGCΔ*cac1502*Δ*upp* Δ*cac3535* strains. The transformation efficiency of MGCΔ*cac1502*Δ*upp*Δ*cac3535* for unmethylated pCons2.1 was ~eightfold higher than that of MGCΔ*cac1502* (Table 2).

**Table 2:** Transformation efficiencies of MGC*Δcac1502* and MGC*Δcac1502*Δ*upp*Δ*cac3535* for unmethylated pCons2.1

	MGCΔ*cac1502*	MGC Δ*cac1502*Δ*upp* Δ*cac3535*
Unmethylated pCons2.1	0.79 (±0.24) × 10^4^	6.1 (±3.2) × 10^4^

Values are expressed in number of transformants per μg DNA

Mean values and standard deviations from three independent experiments are given

25 ng pCons2.1 was used in each experiment

### Determination of the recognition sequence of *Cac824*II encoded by *CA_C3535*

**Fig. 4:**
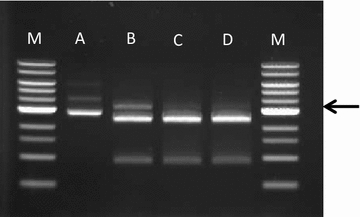
Digestion properties of recombinant *Cac*824II as compared to commercial *Acu*I (New England Biolabs). 250 ng of unmethylated pCONS2.1 plasmid were incubated for 1 h at 37 °C in a reaction volume of 20 µL containing 50 mM potassium acetate, 20 mM Tris–acetate (pH 7.9), 10 mM Magnesium acetate, 100 µg/mL BSA and 0.04 mM S-adenosyl-methionine with (*A*) No enzyme, (*B*) purified *Cac*824II (50 µg), (*C*) *Acu*I (5U), and (*D*) purified *Cac*824II (50 µg) + *Acu*I (5U). *Lanes* *M*, 1 kb DNA ladder (0.5–10 kbp, NEB). Reactions products were electrophoresed on a 0.8 % agarose gel. An *arrow* indicates the incomplete digestion product remaining after incubation with *Cac*824II

*CA_C3535* encoded a 993 amino acid protein with a calculated molecular mass of 116,842 Da. The amino acid sequence analysis revealed high similarities with two restriction endonucleases: *Acu*I from *Acinetobacter calcoaceticus* SRW4 [13] and *Eco*57I from *E. coli* RFL57 [14] with 44 and 46 % identity, respectively. Both enzymes belong to the IIg family of restriction enzymes and possess both a restriction and methylase activity. To heterologously express the *Acu*I-encoding gene in *E. coli* [13], it was necessary to first express the *Acu*IM methylase-encoding gene because the methylase activity of *Acu*I was not sufficient to protect DNA against its restriction activity. We applied the same strategy for the expression of *CA_C3535*-encoding *Cac824*II: we cloned into the pSOS2K2 gene and expressed in *E. coli* the *CA_C3534* gene that encodes a putative methylase and that is located immediately downstream of *CA_C3535* gene in the *C. acetobutylicum* chromosome. The pSC-CAC3534 plasmid expressing *CA_C3534* has three *Acu*I recognition sites, but when we tried to digest it with *Acu*I, it was completely protected from the activity of this enzyme. To express, purify and determine the recognition sequences of *Cac824*II, we cloned *CA_C3535*in the pPAL vector using the *E. coli* BL21-AI cells containing the pSC-CAC3534 plasmid as host. The *Cac824*II endonuclease was purified, and its activity towards unmethylated pCons2.1 in the presence of SAM was determined. *Acu*I recognizes the 5′-CTGAAG-3′ sequence and cuts the pCons2.1 plasmid two times, resulting in two fragments of 2411- and 882-bp. Figure 4 shows that *Cac824*II gives the same restriction pattern as *Acu*I. To confirm that the *Acu*I and *Cac824*II recognition sequences were identical, pCons2.1 was digested by 50 μg of *Cac824*II in the presence of 1 U of *Acu*I. Figure 4 shows that the restriction pattern was unchanged, which definitively confirms that *Acu*I and *Cac824*II are isoschizomers.

### Deletion of the *ctfAB* genes in the MGCΔ*cac1502*Δ*upp*Δ*cac3535* to create a strain no longer producing acetone

**Fig. 5:**
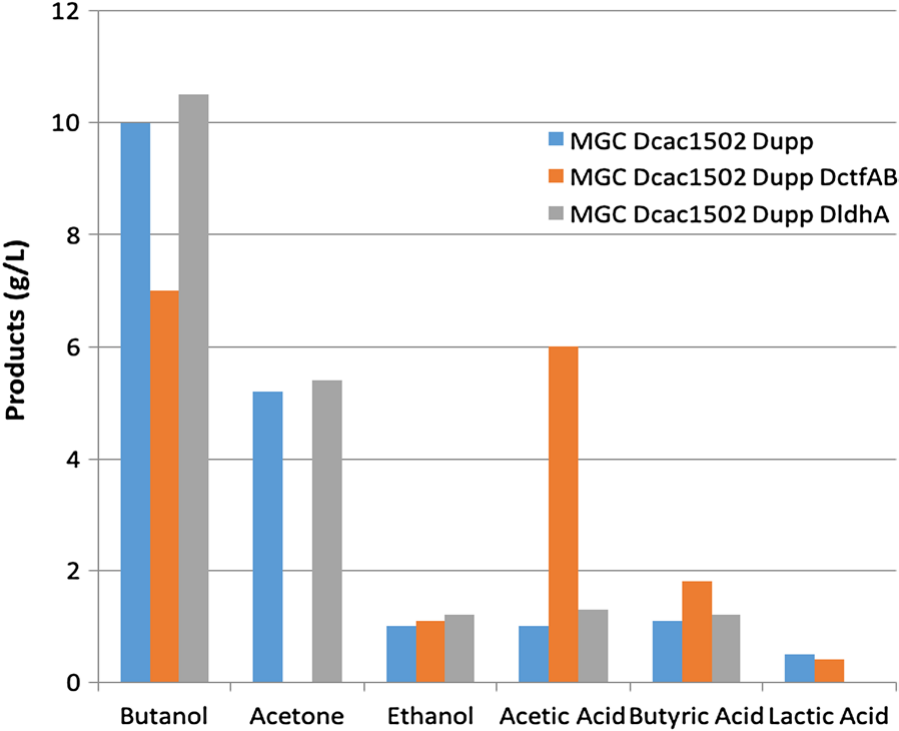
Solvent and acid production of MGCΔ*cac1502* Δ*upp* Δ*cac3535,* MGCΔ*cac1502* Δ*upp* Δ*cac3535*Δ *ctfAB::Em* ^*R*^, and MGCΔ*cac1502* Δ*upp* Δ*cac3535*Δ *ldhA::Em* ^*R*^ in batch culture at pH 4.5 in SM medium

The *ctfAB* genes (*CA_P0163*-*CA_P0164*) located on the pSOL1 megaplasmid encodes an acetoacetyl-CoA:acyl CoA-transferase involved in the first specific step of acetone formation [15]. To delete *ctfAB*, the *ctfAB* replacement cassette was cloned into the *Bam*HI site of the pCons::upp to generate the plasmid pREPctfAB::upp. The plasmid pREPctfAB::upp was used to transform the *C. acetobutylicum* MGCΔ*cac1502*Δ*upp*Δ*cac3535* strain by electroporation without previous in vivo methylation and cell containing the plasmid were selected on RCA plate with erythromycin at 40 µg/ml. To select for Δ*ctfAB::Em*^*R*^ integrants that lost the pREPctfAB::upp plasmid, erythromycin- and 5-FU-resistant clones were selected on RCA plates containing erythromycin and 5-FU from 100 μl of a liquid culture of the MGC*cac1502*Δ*upp*Δ*cac3535* (pREPctfAB::upp) strain. Approximately 500 colonies were obtained, and 50 of them were replica plated on both RCA with erythromycin and RCA with thiamphenicol. Most of the clones (90 %) were resistant to erythromycin and sensitive to thiamphenicol. Four clones were checked by PCR analysis (with primers CTF-0 and CTF-5 located outside of the *ctfAB* replacement cassette and primers CTF-D and CTF-R located inside of *ctfAB*). All four clones had the correct phenotype, and one of the clones was selected as the MGC Δ*cac1502*Δ*upp*Δ*cac3535*Δ*ctfAB::mls*^*R*^ strain. The fermentation profile of this strain was compared to the MGC Δ*cac1502*Δ*upp*Δ*cac3535* control strain during batch fermentation at pH 4.5 (Fig. 5). The production of acetone was totally abolished but the production of acetic acid was increased more than sixfold while butyric acid was only slightly increased, proving that the acetoacetyl-CoA:acyl CoA-transferase is mainly involved in the consumption of acetic acid.

### Deletion of the *ldhA* gene in the MGCΔ*cac1502*Δ*upp*Δ*cac3535* to create a strain no longer producing lactate

The *ldhA* genes (*CA_C0267*) encodes a lactate dehydrogenase involved in the last step of l-lactate formation [15]. To delete *ldhA*, the *ldhA* replacement cassette was cloned into the *Bam*HI site of the pCons::upp to generate the plasmid pREPldhA::upp. The plasmid pREPldhA::upp was used to transform the *C. acetobutylicum* MGCΔ*cac1502*Δ*upp*Δ*cac3535* strain by electroporation without previous in vivo methylation and cell containing the plasmid were selected on RCA plate with erythromycin at 40 µg/ml. To select for Δ*ldhA::Em*^*R*^ integrants that lost the pREPldhA::upp plasmid, erythromycin- and 5-FU-resistant clones were selected on RCA plates containing erythromycin and 5-FU from 100 μl of a liquid culture of the MGC*cac1502*Δ*upp*Δ*cac3535* (pREPldhA::upp) strain. Approximately 500 colonies were obtained, and 50 of them were replica plated on both RCA with erythromycin and RCA with thiamphenicol. Most of the clones (80 %) were resistant to erythromycin and sensitive to thiamphenicol. Four clones were checked by PCR analysis (with primers LDH-0 and LDH-5 located outside of the *ldhA* replacement cassette and primers LDH-D and LDH-R located inside of *ldhA*). All four clones had the correct phenotype, and one of the clones was selected as the MGC Δ*cac1502*Δ*upp*Δ*cac3535*Δ*ldhA::mls*^*R*^ strain. The fermentation profile of this strain was compared to the MGC Δ*cac1502*Δ*upp*Δ*cac3535* control strain during batch fermentation at pH 4.5 (Fig. 5). The production of l-lactate was totally abolished proving that *ldhA* encodes the main l-lactate dehydrogenase of *C. acetobutylicum*.

## Discussion

We developed a simple and efficient method to create mutations in the *Clostridium acetobutylicum* chromosome. This method is based on the use of (1) a replicative plasmid, (2) a deletion cassette containing both DNA sequences with homology to the flanking region of the target gene (to delete it) and an antibiotic resistance gene surrounded by FRT sequences (as an excisable marker), and (3) the *upp* gene, which encodes the uracil–phosphoribosyl-transferase, as a counter-selectable marker.

A plasmid that replicates via a rolling circle mechanism was more efficient in terms of double cross over frequency than a plasmid that replicates through a theta mechanism. This result is in agreement with previous findings in *Bacillus subtilis* showing that plasmid replication through a rolling circle mechanism favors recombination between homologous sequences [16, 17].

The deletion cassette can be rapidly constructed through a three-step procedure using pre-constructed building blocks. After a fusion PCR and TOPO cloning of the product, a predesigned antibiotic resistance gene surrounded by two FRT sites in direct repeats is inserted. The *upp* gene is located on the plasmid outside of the deletion cassette. This allows the positive selection of clones that have lost the plasmid and integrated the deletion cassette by a double recombination event. We demonstrate here that this event occurs at a frequency of 10^−5^, which means that without the selection procedure, it would be much more difficult to isolate the correct deletion mutant by replica plating alone. Once the deletion cassette is integrated into the chromosome, the expression of the *flp* recombinase allows (1) the excision of the antibiotic marker for a clean in-frame deletion of the targeted gene (without polar effect) and (2) consecutive gene deletions. Such a strategy was previously applied to marker-less gene deletion in *E. coli* [18] and *Mycobacterium smegmatis* [19]. The plasmid expressing the FLP recombinase-encoding gene was further improved by coexpressing the *upp* gene to use it as a positive selection for the plasmid loss after excision of the *MLS*^*R*^ marker. A similar tool was developed by Al-Hinai et al. [5] using a plasmid (1) that expresses the FLP recombinase-encoding gene and (2) that has an inducible segregational instability to promote the plasmid loss.

In this study *Cac824*II (encoded by *CA_C3535*), the second type II restriction enzyme of *C. acetobutylicum* predicted by REBASE [20], was biochemically characterized and it was demonstrated that it is an isoschizomer of *Acu*I [13] recognizing the 5′-CTGAAG-3′ sequence. It was also shown that *Cac824*II methylase (encoded by *CA_C3534*) protects DNA against restriction by *Cac824*II and *Acu*I by probably methylating one of the adenine in the 5′-CTGAAG-3′ sequence. Two *Cac824*II restriction sites are present (in the *ampR* gene and in the colE1 origin of replication) in most the shuttle vector use to transform *C. acetobutylicum* and it was then justified to construct a marker-less strain deleted from CA_C3535. The transformation efficiency of MGCΔ*cac1502*Δ*upp*Δ*cac3535* for unmethylated pCons2.1 was much higher (~eightfold higher) than that of MGC*Δcac1502* and it will be an interesting strain to develop new genetic tools based on suicide vectors [20].

The restriction-less marker-less strain and the method was successfully used to delete two genes (*ctfAB*) on the pSOL1 megaplasmid and one gene (*ldhA*) on the chromosome to get strains no longer producing acetone or l-lactate. This work demonstrate that (1) *ctfAB* encode an acetoacetyl-CoA:acetate CoA-transferase that coupled acetone formation to acetate consumption and (2) *ldhA* encodes the main lactate dehydrogenase in *C. acetobutylicum* although a second gene *ldhB* (*CA_C3552*) is also present [15]. A strain with clostron inactivated *ctfAB* genes was previously constructed [21]. From the physiological analysis of this mutant and with the help of a mathematical model [22], it was demonstrated that butyrate was mainly reconsumed by the phosphotransbutyrylase-buyrate-kinase pathway and not by the acetoacetyl-CoA:acetate CoA-transferase in agreement with the data presented in our study.

## Conclusions

The restriction-less, marker-less strain and the genome modification method presented here become simple and convenient tools that are useful for research groups involved in functional genomic studies of *C. acetobutylicum* and for further metabolic engineering of this strain to produce bulk chemicals and biofuel. As a demonstration of the efficiency of the method, we constructed two strains unable to produce l-lactate or acetone. Furthermore, this method was successfully used by the Metabolic Explorer Company to develop and patent an industrial recombinant strain of *C. acetobutylicum* for *n*-butanol production [23] at high yield.

## Methods

### Bacterial strain, plasmids and oligonucleotides

The bacterial strain and plasmids used in this study are listed in Table 3. The specific oligonucleotides used for PCR amplification were synthesized by Eurogentec (Table 4).

**Table 3:** Bacterial strains and plasmids used in this study

Strain or plasmid	Relevant characteristics^a^	Source or reference^b^
Bacterial strains		
*E. coli*		
TOP10		Invitrogen
ER2275	RecA^−^ McrBC^−^	NEB
*C. acetobutylicum*		
ATCC824	Wild type	ATCC
MGC*Δcac1502*	Δ*CA_C1502*	This study
MGC*Δcac1502*Δ*upp*	Δ*CA_C 1502*Δ *CA_C 2879*	This study
MGC*Δcac1502*Δ*upp*Δ*cac3535*	Δ*CA_C 1502*Δ*CA_C 2879*Δ*CA_C 3535*	This study
MGC*Δcac1502*Δ*upp*Δ*cac3535*Δ*ctfAB*	Δ*CA_C 1502*Δ*CA_C 2879*Δ*CA_C 3535*Δ*CA_P0162*-*3*	This study
MGC*Δcac1502*Δ*upp*Δ*cac3535*Δ*ldhA*	Δ*CA_C 1502*Δ*CA_C 2879*Δ*CA_C 3535*Δ*CA_C 0267*	This study
Plasmids		
pAN1	Cm^r^, φ3TI, p15A origin	[27]
pKD4	Ap^r^ Km^r^	[18]
pETSPO	Cm^r^ MLS^r^	[4]
pUC18	Ap^r^	Fermentas
pUC18-FRT-MLS2	Ap^r^ MLS^r^	This study
pCons2-1	Cm^r^	This study
pCR-BluntII-TOPO	Zeo^r^ Km^r^	Invitrogen
pCIP2-1	Cm^r^	This study
pREPcac15	Cm^r^ MLS^r^ Δ*CA_C1502*	This study
pCIPcac15	Cm^r^ MLS^r^ Δ*CA_C1502*	This study
pREPupp	Cm^r^ MLS^r^ Δ*upp*	This study
pCP20	Ap^r^ Cm^r^ *FLP*	[29]
pSOS95	Ap^r^ MLS^r^, acetone operon, *repL* gene, ColE1 origin	[32]
pCLF1	Cm^r^ *FLP*	This study
pCR4-TOPO-Blunt	Ap^r^ Km^r^	Invitrogen
pCons::upp	Cm^r^ MLS^r^ *upp*	This study
pREPCAC3535::upp	Cm^r^ MLS^r^ *upp* Δ*CA_C3535*	This study
pREPctfAB::upp	Cm^r^ MLS^r^ *upp* Δ*ctfAB*	This study
pREPldhA::upp	Cm^r^ MLS^r^ *upp* Δ*ldhA*	This study

^a^ *RecA* ^*−*^ homologous recombination abolished, *McrBC* ^*−*^ lacking methylcytosine-specific restriction system, *Cm* ^*r*^ chloramphenicol resistance, *Ap* ^*r*^ ampicillin resistance, *MLS* ^*r*^ macrolide lincosamide and streptogramin B resistance, *Zeo* ^*r*^ zeomycin resistance, *φ3TI* φ3TI methyltransferase, *repL* Gram-positive origin of replication from pIM13

^b^ *NEB* New England BioLabs, *ATCC* American Type Culture Collection (Rockville, MD)

**Table 4:** Oligonucleotides used for PCR amplifications

Primer name	Oligonucleotide sequence
PKD4.1	ct*ggcgcc*ctgagtgcttgcggcagcgtgagggg
PKD4.2	ag*cccgggg*atctcatgctggagttcttcgccc
FRT-MLSR-F	tac*aggcct*tgagcgattgtgtaggctggagc
FRT-MLSR-R	aac*aggcct*gggatgtaacgcactgagaagccc
PCONSAccI	ccggggtaccgtcgacctgcagcc
PCONS*Eco*RI	gaattccgcgagctcggtacccggc
ORI3-D	ccatcgatgggggtcatgcatcaatactatcccc
ORI4-R	gcttccctgttttaatacctttcgg
FLP1-D	aaaa*ggatcc*aaaaggagggattaaaatgccacaatttggtatattatgtaaaacaccacct
FLP1-R	aaat*ggcgcc*gcgtacttatatgcgtctatttatgtaggatgaaaggta
REP-UPP-F	aaaacagctgggaggaatgaaataatgagtaaagttacac
REP-UPP-R	aaaacagctgttattttgtaccgaataatctatctccagc
CAC 1	aaa*ggatcc*atgcacactcataaatttactgtaggaagtctg
CAC 2	gggg*aggcct*aaaaaggggggtcccaaataatatttgccatagtaaccacc
CAC 3	cccccttttt*aggcct*cccctcgaacttattagaatgattaagattccgg
CAC 4	aaa*ggatcc*tcattaaatttcctccattttaagcctgtc
CAC 0	gtgatataattttcctttaaatggaggaggatctg
CAC 5	gccgttaatagacattataattccattggc
CAC-D	gaattcttaaaaatatttggatcattaagcgg
CAC-R	gttgtattggaatctttgttattatttctccc
UPP 1	aaaa*ggatcc*tcctgatctattaattcttgatgaaccc
UPP 2	gggg*aggcct*aaaaagggggattgcataaataaaaagggctgaaaaataaatttcag
UPP 3	cccccttttt*aggcct*ccccttatttcattcctccattgtattttttttctatttg
UPP 4	aaaa*ggatcc*gctattatgaataggttaaataagtcagctgg
UPP 0	aatacaagcaaagagaataggctatgtgcc
UPP 5	aatacaagcaaagagaataggctatgtgcc
UPP-D	ggcatatgaagtaacaagagaaatgcagc
UPP-R	ataatctatctccagcatctccaagacc
RM3535 1	aaaa*ggatcc*gcagctttctggaaggactacggcg
RM3535 2	gggg*aggcct*aaaaagggggcatttacttatggtacggttcacccc
RM3535 3	cccccttttt*aggcct*ccccgtctttaaaaagtaatttatcaaaggcatcaaggc
RM3535 4	aaaa*ggatcc*ctaactctctaaacgttacaatagtaatgcgc
RM3535 0	cacattgtcatttataaaagtccctaggg
RM3535 5	gtagtaattccaacttcaactcttgccac
RM3535-D	cttagaatagctgatattgcttgcgg
RM3535-R	agcatctctcttaatgattctccgg
CTF1	aaaaggatcccagacactataatagctttaggtggtacccc
CTF2	ggggaggcctaaaaagggggattataaaaagtagttgaaatatgaaggtttaaggttg
CTF3	ccccctttttaggcctccccatatccaatgaacttagacccatggctg
CTF4	aaaaggatccgtgttataatgtaaatataaataaataggactagaggcg
CTF0	taccaccttctttcacgcttggctgcgg
CTF5	tatttaaagaggcattatcaccagagcg
LDH1	aaaaggatccgctttaaaatttggaaagaggaagttgtg
LDH2	ggggaggcctaaaaagggggttagaaatctttaaaaatttctctatagagcccatc
LDH3	ccccctttttaggcctccccggtaaaagacctaaactccaagggtggaggctaggtc
LDH4	aaaaggatcccccattgtggagaatattccaaagaagaaaataattgc
LDH0	cagaaggcaagaatgtattaagcggaaatgc
LDH5	cttcccattatagctcttattcacattaagc
Cac3535-d-*Spe*I	aaa*actagt*atgaatgatattaaaatagctttgaaaaaattggttgac
Cac3535-R-*Bam*HI	aaaa*ggatcc*ctacaaattatatatatctgttaccaatgcctc

Restriction sites are in italic

### Culture and growth conditions

*C. acetobutylicum* was maintained as spores in synthetic medium (SM) as previously described [24, 25] Spores were activated by heat treatment at 80 °C for 15 min. All *C. acetobutylicum* strains were grown in anaerobic conditions at 37 °C in SM, in *Clostridium* growth medium (CGM) [26] in 2YTG [27] or in reinforced clostridial medium (RCM) (Fluka). Solid media were obtained by adding 1.5 % agar to the liquid media. Media were supplemented, when required, with the appropriate antibiotic in the following concentrations: for *C. acetobutylicum*, erythromycin at 40 µg/ml and thiamphenicol at 50 µg/ml; for *E. coli*, erythromycin at 200 µg/ml and chloramphenicol at 30 µg/ml. Transformations of *C. acetobutylicum* were conducted by electroporation, as previously described [11]. 5-FU was purchased from Sigma, and stock solutions were prepared in DMSO (dimethyl sulfoxide).

### DNA manipulation techniques

Total genomic DNA from *C. acetobutylicum* was isolated as previously described [27]. Plasmid DNA was extracted from *E. coli* with the QIAprep kit (Qiagen, France). Pfu DNA Polymerase (Roche) was used to generate PCR products for cloning, and Taq Polymerase (New England BioLabs) was used for screening colonies by PCR with standard PCR protocols employed for all reactions. DNA restriction and cloning were performed according to standard procedures [28]. Restriction enzymes and Quick T4 DNA ligase were obtained from New England BioLabs (Beverly, MA) and were used according to the manufacturer’s instructions. DNA fragments were purified from agarose gels with the QIAquick gel purification kit (Qiagen, France).

### Construction of pUC18-FRT-MLS2

Inverse PCR was performed using the pKD4 plasmid [18] as a template and oligonucleotides PKD4.1 and PKD4.2 as primers to amplify the plasmid region with the FRT sites but without the kanamycin resistance marker. This blunt end fragment was later ligated to the *MLS*^*r*^ gene obtained after a *Hind*III digestion of the pETSPO plasmid [4] and Klenow treatment. The corresponding plasmid (pKD4-Ery1) was then used as a template to amplify by PCR the macrolide lincosamide streptogramin B resistance (*MLS*^*r*^) gene, functional in *Clostridia* and flanked by two FRT sites and two *Stu*I sites, using the oligonucleotides FRT-MLSR-F and FRT-MLSR-R as primers. This fragment was directly cloned into the *Sma*I digested pUC18 to generate the pUC18-FRT-MLS2 plasmid.

### Construction of pCons2.1

Inverse PCR was performed using the pETSPO plasmid [4] as a template and oligonucleotides PCONSAccI (mutating a *Bam*HI site) and PCONS*Eco*RI as primers. The PCR product, containing a pIMP13 *B. subtilis* origin of replication functional in *Clostridia* (rolling circle mechanism of replication) and a *catP* gene conferring resistance to thiamphenicol was phosphorylated and ligated to yield the pCons0 plasmid. This plasmid was then digested with *Bam*HI to remove the *spoA* cassette, and the DNA fragment was purified and ligated to generate the pCons2-1 plasmid.

### Construction of pCIP2-1

The pIMP13 origin of replication from pCons2-1 was replaced by the origin of replication of the pSOL1 megaplasmid. The origin of replication of pSOL1 was amplified by PCR using *C. acetobutylicum* total DNA as a template and oligonucleotides ORI3-D and ORI3-R as primers. This PCR product was cloned into the pCR-BluntII-TOPO vector, and the resulting plasmid was digested by *Eco*RI to obtain the 2.2 kb *Eco*RI fragment containing the origin of replication of pSOL1. The pCons2-1 plasmid was digested by *Eco*RI, and the 2.4 kb fragment was ligated to the 2.2 kb *Eco*RI fragment to generate the plasmid pCIP2-1.

### Construction of pREPcac15

Two DNA fragments surrounding *cac*1502 were amplified by PCR using *C. acetobutylicum* total DNA as the template and two pairs of oligonucleotides as primers. Using the primers pairs CAC 1 and CAC 2 or CAC 3 and CAC 4, 1493 and 999-bp DNA fragments were obtained, respectively. Both primers CAC 1 and CAC 4 introduce a *Bam*HI site, whereas primers CAC 2 and CAC 3 have complementary 5′ extended sequences that introduce a *Stu*I site. DNA fragments CAC 1–CAC 2 and CAC 3–CAC 4 were joined in a PCR fusion with primers CAC 1 and CAC 4, and the resulting fragment was cloned into the pCR4-TOPO-Blunt vector to generate pTOPO::cac15. At the unique *Stu*I site of pTOPO::cac15, the 1372-bp *Stu*I fragment of pUC18-FRT-MLS2 carrying the antibiotic resistance *MLS*^*r*^ gene with FRT sequences on both sides was introduced. The *cac*1502 replacement cassette obtained after *Bam*HI digestion of the resulting plasmid was cloned into the *Bam*HI site of the pCons2-1 to generate the plasmid pREPcac15.

### Construction of pCIPcac15

The *cac*1502 replacement cassette above was cloned into the *Bam*HI site of the pCIP2-1 to generate the plasmid pCIPcac15.

### Construction of pREPupp

Two DNA fragments upstream and downstream of *cac*2879 were amplified by PCR using total DNA from *C. acetobutylicum* as the template and two pairs of oligonucleotides as primers. With the primer pairs UPP 1–UPP 2 and UPP 3–UPP 4, 1103- and 1105-bp DNA fragments were obtained, respectively. Both primers UPP 1 and UPP 4 introduce a *Bam*HI site, whereas primers UPP 2 and UPP 3 have 5′ extended sequences that introduce a *Stu*I site. DNA fragments UPP 1–UPP 2 and UPP 3–UPP 4 were joined in a PCR fusion with primers UPP 1 and UPP 4, and the resulting fragment was cloned into pCR4-TOPO-Blunt vector to generate pTOPO::upp. At the unique *Stu*I site of pTOPO::upp, the 1372-bp *Stu*I fragment of pUC18-FRT-MLS2 carrying the antibiotic resistance MLS^r^ gene with FRT sequences on both sides was introduced. The *upp* replacement cassette obtained after *Bam*HI digestion of the resulting plasmid was cloned into the *Bam*HI site of the pCons2-1 to generate the plasmid pREPupp.

### Construction of pCLF1

The *FLP1* gene was amplified by PCR using the pCP20 plasmid [29] as a template and oligonucleotides FLP1-D and FLP1-R as primers. These primers introduced *Bam*HI and *Sfo*I restriction sites on the ends of the PCR product. After a *Bam*HI–*Sfo*I double digestion, the PCR product was cloned into the *Bam*HI–*Sfo*I sites of the pSOS95 expression vector to generate the pEX-FLP1 plasmid. The 1585-bp *Sal*I fragment of pEX-FLP1 containing the *FLP1* expression cassette was cloned into the *Sal*I site of pCons2-1 to generate the pCLF1 plasmid.

### Construction of pCons::upp

The *upp* gene with its own ribosome binding site (RBS) was amplified by PCR from *C. acetobutylicum* total DNA with the oligonucleotides REP-UPP-F and REP-UPP-R as primers. The 664-bp PCR product was digested by *Pvu*II and was cloned into pCons2.1, digested by *Bcg*I and treated with T4 DNA polymerase to generate the pCons::*upp* plasmid. In this way, the *upp* gene was located just downstream of the *catP* gene to construct an artificial operon with *upp* expressed under the control of the *catP* promoter.

### Construction of pREPcac35::upp

Two DNA fragments upstream and downstream of *CA_C3535* were amplified by PCR using the total DNA from *C. acetobutylicum* as a template and two pairs of oligonucleotides as primers. With the primer pairs RM3535 1 and RM3535 2 or RM3535 3 and RM3535 4, 1044- and 938-bp DNA fragments were obtained, respectively. Both primers RM3535 1 and RM3535 4 introduce a *Bam*HI site, whereas primers RM3535 2 and RM3535 3 have 5′ extended sequences that introduce a *Stu*I site. DNA fragments RM3535 1-RM3535 2 and RM3535 3-RM3535 4 were joined in a PCR fusion with primers RM3535 1 and RM3535 4, and the resulting fragment was cloned into the pCR4-TOPO-Blunt vector to generate pTOPO::cac3535. At the unique *Stu*I site of pTOPO::cac3535, the 1372-bp *Stu*I fragment of pUC18-FRT-MLS2 carrying the antibiotic resistance *MLS*^r^ gene with FRT sequences on both sides was introduced. The *CA_C3535* replacement cassette obtained after *Bam*HI digestion of the resulting plasmid was cloned into the *Bam*HI site of the pCons::upp to generate the plasmid pREPcac3535::upp.

### Construction of pREPctfAB::upp

Two DNA fragments upstream and downstream of *ctfAB* (*CA_P0162*-*CA_P0163*) were amplified by PCR using the total DNA from *C. acetobutylicum* as a template and two pairs of oligonucleotides as primers. With the primer pairs CTF 1 and CTF 2 or CTF 3 and CTF 4, 1144- and 1138-bp DNA fragments were obtained, respectively. Both primers CTF 1 and CTF 4 introduce a *Bam*HI site, whereas primers CTF 2 and CTF 3 have 5′ extended sequences that introduce a *Stu*I site. DNA fragments CTF 1-CTF 2 and CTF 3-CTF 4 were joined in a PCR fusion with primers CTF 1 and CTF 4, and the resulting fragment was cloned into the pCR4-TOPO-Blunt vector to generate pTOPO::ctfAB. At the unique *Stu*I site of pTOPO::ctfAB, the 1372-bp *Stu*I fragment of pUC18-FRT-MLS2 carrying the antibiotic resistance *MLS*^r^ gene with FRT sequences on both sides was introduced. The *ldhA* replacement cassette obtained after *Bam*HI digestion of the resulting plasmid was cloned into the *Bam*HI site of the pCons::upp to generate the plasmid pREPctfAB::upp.

### Construction of pREPldhA::upp

Two DNA fragments upstream and downstream of *ldhA* (*CA_C0267*) were amplified by PCR using the total DNA from *C. acetobutylicum* as a template and two pairs of oligonucleotides as primers. With the primer pairs LDH 1 and LDH 2 or LDH 3 and LDH 4, 1135- and 1161-bp DNA fragments were obtained, respectively. Both primers LDH 1 and LDH 4 introduce a *Bam*HI site, whereas primers LDH 2 and LDH 3 have 5′ extended sequences that introduce a *Stu*I site. DNA fragments LDH 1-LDH 2 and LDH 3-LDH 4 were joined in a PCR fusion with primers LDH 1 and LDH 4, and the resulting fragment was cloned into the pCR4-TOPO-Blunt vector to generate pTOPO::ldhA. At the unique *Stu*I site of pTOPO::ldhA, the 1372-bp *Stu*I fragment of pUC18-FRT-MLS2 carrying the antibiotic resistance *MLS*^r^ gene with FRT sequences on both sides was introduced. The *ldhA* replacement cassette obtained after *Bam*HI digestion of the resulting plasmid was cloned into the *Bam*HI site of the pCons::upp to generate the plasmid pREPldhA::upp.

### Construction of pCLF::upp

The 1585-bp *Sal*I fragment of pEX-FLP1 containing the *FLP1* expression cassette was cloned into the *Sal*I site of pCons::upp to generate the pCLF::upp plasmid.

#### *Cac*3535 expression and purification

For the general cloning methods of restriction endonuclease genes in *E. coli*, the first step to clone and express the recombinant *CA_C3535* gene into *E. coli* was to pre-protect the host genomic DNA against the restriction activity of the *Cac*3535 bi-functional enzyme. The *CA_C3534* methylase-encoding gene was thus amplified by PCR with Phusion DNA polymerase using *C. acetobutylicum* ATCC824 total genomic DNA as the template and *Cac*3534-d-*Age*I and *Cac*3534-R-*Pvu*I as primers. After digestion with *Age*I and *Pvu*I, the resulting 1748-bp fragment was then cloned into pAH105 [30] a pSC101 derivative, that has been previously digested with *Age*I and *Pac*I, resulting in the pSC-CAC3534 plasmid. In this construct, the *CA_C3534* gene expression was placed under the control of the pGI 1.6 promoter [31].

The *E. coli* BL21-AI strain (Invitrogen) was then transformed by the pSC-CAC3534 plasmid to give the BL21-AI-3534 strain. This strain, with host genomic DNA protected against the restriction activity of the *Cac*3535 bi-functional enzyme, was finally used as the host strain for the *CA_C3535* gene over-expression using the T7-based expression system (see below). The *Cac*3535 protein was expressed in *E. coli* BL21 AI-3534 and was purified using the Profinity eXact Protein Purification System, following the recommendations of the manufacturer (Biorad). The *CA_C3535* gene was amplified by PCR with Phusion DNA polymerase using *C. acetobutylicum* ATCC824 total gDNA as the template and *Cac*3535-d-*Spe*I and *Cac*3535-R-BamHI as primers. The resulting 3002 bp fragment was cloned into the Zero Blunt TOPO vector (Invitrogen) to generate the TOPO-CAC3535 plasmid. After verification by DNA sequencing, the 2988-bp *Spe*I-*Bam*HI fragment from the latter plasmid was then introduced into the pPAL7 vector previously digested with the same enzymes to give the final pPAl-3535-I_2.4 plasmid.

*After transformation, E. coli* BL21-AI-3534 cells harboring the pPAl-3535-I_2.4 plasmid were grown in TB medium in the presence of 50 µg/ml carbenicillin and 100 µg/ml Spectinomycin at 37 °C to an OD550 ~ 0.45 and were then induced with 500 µM IPTG for 4 h at 37 °C. After centrifugation, the cell lysate was obtained by sonicating the resuspended pellet in bind/wash buffer (0.1 M sodium phosphate buffer, pH 7.2).

The tag-free *Cac*3535 protein was prepared using the Profinity eXact protein purification system, according to the standard protocol. After the Profinity Exact mini-spin column was bound by the protein and washed, the proteolytic activity of the affinity matrix was activated by applying two column volumes of room temperature 0.1 M sodium phosphate buffer, pH 7.2, containing 0.1 M sodium fluoride. The column was incubated for 30 min to allow for the cleavage of the tag from the protein; then, the tag-free protein was released from the mini-spin column by centrifugation. The tag-free *Cac*3535 purified protein retains a Thr-Ser linker at its N-Terminus, ensuring optimal binding and cleavage during the purification steps (“Imprecise Fusion protein”).

## Abbreviations

5-FU: 5-fluorouracil
CGM: *Clostridium* growth medium
DMSO: dimethyl sulfoxide
FLP: flippase
FRT: flippase recognition target
*MLS*^*r*^: the macrolide lincosamide streptogramin B resistance gene
PCR: polymerase chain reaction
RBS: ribosome binding site
RCM: reinforced clostridial medium
SM: synthetic medium
*Th*^*R*^: thiamphenicol resistance gene
UPRTase: uracil phosphoribosyl-transferase

## References

[1] Nolling J, Breton G, Omelchenko MV, Makarova KS, Zeng Q, Gibson R, Lee HM, Dubois J, Qiu D, Hitti J (2001). Genome sequence and comparative analysis of the solvent-producing bacterium C*lostridium acetobutylicum*. J Bacteriol.

[2] Green EM, Boynton ZL, Harris LM, Rudolph FB, Papoutsakis ET, Bennett GN (1996). Genetic manipulation of acid formation pathways by gene inactivation in *Clostridium acetobutylicum* ATCC 824. Microbiology.

[3] Green EM, Bennett GN (1996). Inactivation of an aldehyde/alcohol dehydrogenase gene from *Clostridium acetobutylicum* ATCC 824. Appl Biochem Biotechnol.

[4] Harris LM, Welker NE, Papoutsakis ET (2002). Northern, morphological, and fermentation analysis of spo0A inactivation and overexpression in *Clostridium acetobutylicum* ATCC 824. J Bacteriol.

[5] Al-Hinai MA, Fast AG, Papoutsakis ET (2012). Novel system for efficient isolation of *Clostridium* double-crossover allelic exchange mutants enabling markerless chromosomal gene deletions and DNA integration. Appl Environ Microbiol.

[6] Liu CC, Qi L, Yanofsky C, Arkin AP (2011). Regulation of transcription by unnatural amino acids. Nat Biotechnol.

[7] Heap JT, Ehsaan M, Cooksley CM, Ng YK, Cartman ST, Winzer K, Minton NP (2012). Integration of DNA into bacterial chromosomes from plasmids without a counter-selection marker. Nucleic Acids Res.

[8] Heap JT, Pennington OJ, Cartman ST, Carter GP, Minton NP (2007). The ClosTron: a universal gene knock-out system for the genus *Clostridium*. J Microbiol Methods.

[9] Shao L, Hu S, Yang Y, Gu Y, Chen J, Yang Y, Jiang W, Yang S (2007). Targeted gene disruption by use of a group II intron (targetron) vector in *Clostridium acetobutylicum*. Cell Res.

[10] Soucaille P, Figge R, Croux C, Explorer M. Process for chromosomal integration and DNA sequence replacement in *Clostridia*. International Patent Application PCT/EP2006/066997. 2006.

[11] Mermelstein LD, Papoutsakis ET (1993). In vivo methylation in *Escherichia coli* by the *Bacillus subtilis* phage phi 3T I methyltransferase to protect plasmids from restriction upon transformation of *Clostridium acetobutylicum* ATCC 824. Appl Environ Microbiol.

[12] Fabret C, Ehrlich SD, Noirot P (2002). A new mutation delivery system for genome-scale approaches in *Bacillus subtilis*. Mol Microbiol.

[13] Samuelson J, Xu S, O’Loane D, New England Biolabs I. Method for cloning and expression of *Acu*I restriction endonuclease and *Acu*I methylase in *E. coli*. US patent No.7,011,966. 2006.

[14] Janulaitis A, Vaisvila R, Timinskas A, Klimasauskas S, Butkus V (1992). Cloning and sequence analysis of the genes coding for Eco57I type IV restriction-modification enzymes. Nucleic Acids Res.

[15] Yoo M, Bestel-Corre G, Croux C, Riviere A, Meynial-Salles I, Soucaille P (2015). A quantitative system-scale characterization of the metabolism of *Clostridium acetobutylicum*. MBio.

[16] Noirot P, Petit MA, Ehrlich SD (1987). Plasmid replication stimulates DNA recombination in *Bacillus subtilis*. J Mol Biol.

[17] Petit MA, Mesas JM, Noirot P, Morel-Deville F, Ehrlich SD (1992). Induction of DNA amplification in the *Bacillus subtilis* chromosome. EMBO J.

[18] Datsenko KA, Wanner BL (2000). One-step inactivation of chromosomal genes in *Escherichia coli* K-12 using PCR products. Proc Natl Acad Sci USA.

[19] Stephan J, Stemmer V, Niederweis M (2004). Consecutive gene deletions in *Mycobacterium smegmatis* using the yeast FLP recombinase. Gene.

[20] Sillers R, Chow A, Tracy B, Papoutsakis ET (2008). Metabolic engineering of the non-sporulating, non-solventogenic *Clostridium acetobutylicum* strain M5 to produce butanol without acetone demonstrate the robustness of the acid-formation pathways and the importance of the electron balance. Metab Eng.

[21] Cooksley CM, Zhang Y, Wang H, Redl S, Winzer K, Minton NP (2012). Targeted mutagenesis of the *Clostridium acetobutylicum* acetone-butanol-ethanol fermentation pathway. Metab Eng.

[22] Millat T, Voigt C, Janssen H, Cooksley CM, Winzer K, Minton NP, Bahl H, Fischer RJ, Wolkenhauer O (2014). Coenzyme A-transferase-independent butyrate re-assimilation in *Clostridium acetobutylicum*-evidence from a mathematical model. Appl Microbiol Biotechnol.

[23] Soucaille P. Metabolic engineering of *Clostridium acetobutylicum* for enhanced production of n-butanol. International patent application WO2008052973.

[24] Vasconcelos I, Girbal L, Soucaille P (1994). Regulation of carbon and electron flow in *Clostridium acetobutylicum* grown in chemostat culture at neutral pH on mixtures of glucose and glycerol. J Bacteriol.

[25] Peguin S, Goma G, Delorme P, Soucaille P (1994). Metabolic flexibility of *Clostridium acetobutylicum* in response to met. Appl Microbiol Biotechnol.

[26] Wiesenborn DP, Rudolph FB, Papoutsakis ET (1988). Thiolase from *Clostridium acetobutylicum* ATCC 824 and its role in the synthesis of acids and solvents. Appl Environ Microbiol.

[27] Mermelstein LD, Welker NE, Bennett GN, Papoutsakis ET (1992). Expression of cloned homologous fermentative genes in *Clostridium acetobutylicum* ATCC 824. Biotechnology (NY).

[28] Sambrook J, Fritsch EF, Maniatis T. Molecular cloning: a laboratory manual. 2nd ed. NY: Cold Spring Harbor Laboratory Press; 1989.

[29] Cherepanov PP, Wackernagel W (1995). Gene disruption in *Escherichia coli*: TcR and KmR cassettes with the option of Flp-catalyzed excision of the antibiotic-resistance determinant. Gene.

[30] Payne MS, Picataggio SK, Hsu AK, Nair RV, Valle F, Soucaille P, Trimbur DE, Inc GI, Company EIDPDNA. Promoter and plasmid system for genetic engineering. 2012.

[31] Meynial-Salles I, Cervin MA, Soucaille P (2005). New tool for metabolic pathway engineering in *Escherichia coli*: one-step method to modulate expression of chromosomal genes. Appl Environ Microbiol.

[32] Raynaud C, Sarcabal P, Meynial-Salles I, Croux C, Soucaille P (2003). Molecular characterization of the 1,3-propanediol (1,3-PD) operon of *Clostridium butyricum*. Proc Natl Acad Sci USA.

